# Multidisciplinary Treatment, Including Locoregional Chemotherapy, for Merkel-Polyomavirus-Positive Merkel Cell Carcinomas: Perspectives for Patients Exhibiting Oncogenic Alternative Δ exon 6–7 TrkAIII Splicing of Neurotrophin Receptor Tropomyosin-Related Kinase A

**DOI:** 10.3390/ijms21218222

**Published:** 2020-11-03

**Authors:** Stefano Guadagni, Antonietta Rosella Farina, Lucia Annamaria Cappabianca, Michela Sebastiano, Rita Maccarone, Veronica Zelli, Marco Clementi, Alessandro Chiominto, Gemma Bruera, Enrico Ricevuto, Giammaria Fiorentini, Donatella Sarti, Andrew Reay Mackay

**Affiliations:** 1 Department of Applied Clinical Sciences and Biotechnology, University of L’Aquila, 67100 L’Aquila, Italy; antonietta.farina@univaq.it (A.R.F.); luciaannamaria.cappabianca@univaq.it (L.A.C.); michela.sebastiano@graduate.univaq.it (M.S.); rita.maccarone@univaq.it (R.M.); veronica.zelli@univaq.it (V.Z.); marco.clementi@univaq.it (M.C.); alessandro.chiominto@univaq.it (A.C.); gemma.gbb@gmail.com (G.B.); enrico.ricevuto@univaq.it (E.R.); andrewreay.mackay@univaq.it (A.R.M.); 2 Department of Onco-Hematology, Azienda Ospedaliera “Ospedali Riuniti Marche Nord”, 61122 Pesaro, Italy; g.fiorentini2020@gmail.com (G.F.); d.sarti@fastwebnet.it (D.S.)

**Keywords:** Merkel cell carcinoma, Merkel cell polyomavirus, MCPyV T-antigen, tropomyosin tyrosine kinase receptor, Δ exon 6–7 TrkAIII, isolated pelvic and limb perfusion, larotrectinib, entrectinib

## Abstract

Merkel cell carcinomas (MCCs) are rare, aggressive, cutaneous neuroendocrine tumours, approximately 80% of which are caused by the genomic integration of Merkel cell polyomavirus (MCPyV). MCPyV-positive MCCs carry poor prognosis in approximately 70% of cases, highlighting the need for greater understanding of the oncogenic mechanisms involved in pathogenesis, progression and post-therapeutic relapse, and translation into novel therapeutic strategies. In a previous pilot study, we reported a potential relationship between *MCPyV* gene expression and oncogenic alternative Δ exon 6–7 TrkAIII splicing in formalin-fixed paraffin-embedded (FFPE) MCC tissues from a 12-patient cohort of >90% MCPyV-positive MCCs, diagnosed at San Salvatore Hospital, L’Aquila, Italy, characterising a new MCC subgroup and unveiling a novel potential MCPyV oncogenic mechanism and therapeutic target. This, however, could not be fully verified due to poor RNA quality and difficulty in protein extraction from FFPE tissues. Here, therefore, we extend our previous observations to confirm the relationship between MCPyV and oncogenic alternative Δ exon 6–7 TrkAIII splicing in fresh, nonfixed, MCPyV-positive MCC metastasis by detecting sequence-verified RT-PCR products, including full-length Δ exon 6–7 TrkAIII, and by Western blot detection of a 100 kDa TrkA protein isoform of identical size to 100 kDa Δ exon 6–7 TrkAIII expressed by stable transfected SH-SY5Y cells. We also report that in three MCC patients submitted for multidisciplinary treatment, including locoregional chemotherapy, MCPyV large T-antigen mRNA expression, Δ exon 6–7 TrkAIII mRNA expression and intracellular indirect immunofluorescence (IF) TrkA and phosphorylation protein isoform(s) immunoreactivity in FFPE tissues were not reduced in postchemotherapeutic-relapsed MCCs compared to pretherapeutic MCCs, extending the possible roles of this novel potential MCPyV oncogenic mechanism from MCC pathogenesis to post-therapeutic relapse and progression. Detection of alternative Δ exon 6–7 TrkAIII splicing in MCC, therefore, not only characterises a new MCPyV-positive MCC subgroup and unveils a novel potential MCPyV oncogenic mechanism but also identifies patients who may benefit from inhibitors of MCPyV T-antigen and/or TrkAIII expression or clinically approved Trk kinase inhibitors such as larotrectinib or entrectinib, which are known to inhibit activated TrkA oncogenes and to elicit durable responses in TrkA-fusion oncogene-driven cancers, supporting the call for a large-scale multicentre clinical study.

## 1. Introduction

Merkel cell carcinomas (MCCs) are rare, highly aggressive neuroendocrine skin cancers, the incidence of which is increasing in North America, Europe and Australia. Etiological MCC risk factors include Merkel cell polyomavirus (MCPyV) infection, with genomic integration that accounts for approximately 80% of cases, immunosuppression, ultraviolet (UV)-induced mutation and age greater than 65 years [1,2,3,4,5]. Current first-line treatments for primary and locoregional MCCs include surgical excision with wide margins, followed by radiation therapy (RT) to reduce locoregional recurrence [6]. Recommendations, subject to clinical and/or microscopic lymph node status, also include sentinel lymph node biopsy (SNB) with complete lymph node dissection (CLND) and RT of the nodal basin, where appropriate [6]. Adjuvant RT for MCC is recommended in National Comprehensive Cancer Network (NCCN) guidelines [6] and employed routinely in the USA, although significant differences in regional recurrence rates and overall survival have not been demonstrated [7]. RT is also under consideration for the management of in-transit MCC metastases (stage IIIB) when surgery alone cannot contain the disease or ensure clear margins. However, results for this strategy are inconclusive, nonrandomised and uncontrolled [8].

Current therapeutic strategies for MCCs result in overall 3-year survival rates of approximately 39%, and 26% to 60% of patients submitted for surgery, with or without adjuvant RT, represent with local and locoregional recurrences within two years [9]. For patients with advanced locoregional disease, there is currently no specific multidisciplinary therapeutic consensus [10], and patients with stage IV disease are routinely treated with systemic platinum-based etoposide, anthracycline and taxane regimens, alone or in various combinations. Unfortunately, these treatments invariably result in brief responses [11], highlighting the urgent need for alternative therapeutic strategies. Within this context, phase II clinical trials with novel immune checkpoint inhibitors elicit sustained responses in patients with MCPyV- and UV-induced metastatic MCCs [12,13,14]. Pembrolizumab-programmed cell death protein 1 (PD-1) immune checkpoint inhibitor has been reported to elicit a 56% response rate in a 26-patient MCC cohort [12] and avelumab-programmed death-ligand 1 (PD-L1) inhibitor a 31.8% response rate in a heavily pretreated 88-patient MCC cohort, with a higher response rate in patients not previously exposed to chemotherapy [13]. Promising anecdotal antitumor effects have also been reported for the novel targeted vascular endothelial growth factor receptor (VEGFR) tyrosine kinase inhibitors pazopanib (4 patients) and cabozantinib (1 patient) [15] for direct targeting of MCC cell surface antigens, for an antibody raised against the epithelial cell adhesion molecule-1/cluster of differentiation CD326, and for the cytokines tumour necrosis factor (TNF) and gamma interferon (INFγ) [16]. In contrast, the combination of surgical intervention with locoregional chemotherapeutic procedures has largely been overlooked for treating locoregionally advanced MCCs, with or without distant metastases, despite routine use over the past 20 years as integrated approaches for local disease control and limb preservation in highly specialised American [17,18,19,20,21], European [22,23,24] and Australian [25] centres.

Locoregional chemotherapeutic procedures include (i) isolated limb infusion (ILI) [19,20,21,25], (ii) isolated limb perfusion (ILP) [17,18,19,22,23], and (iii) isolated pelvic and limb perfusion (IPLP) for patients with pelvic and/or inguinal involvement (this study and [24]). All three procedures incorporate technologically controlled extracorporeal blood circulation. ILP is performed under conditions of oxygenation, high flow rates (150–1000 mL/min), circuit hyperthermia to maintain tissue normothermia and requires specialised surgical skill, whereas ILI and IPLP are executed under conditions of hypoxia, low flow-rates (50–150 mL/min) and mild circuit hyperthermia to maintain tissue normothermia, with the option of chemofiltration, and can be performed not only by surgeons but also percutaneously by interventional radiologists [26,27]. In a literature review and despite the absence of standardised therapeutic strategies, 16 ILP and 16 ILI cases in the USA [18,25] and 10 IPL and 3 IPLP cases in Europe (this study and [23,24]) have all employed the nitrogen mustard-like, DNA-alkylating chemotherapeutic agent Melphalan (this study and [18,23,24,25]) In these studies, ILP elicited an impressive 90% overall response rate, associated with 5 months median progression-free survival and 50 months median overall survival [18,23], which was comparable to the approximately 80% overall response rate elicited by ILI, reported without median progression-free survival and overall survival data [18,25]. This suggests that the ILI procedure, which is less complex and associates with fewer complications, should be the preferred procedure for local disease control and limb preservation in advanced-stage limb-localised MCC [25]. On the other hand, IPLP is currently the only appropriate procedure for the locoregional chemotherapeutic management of advanced-stage MCCs that are localised to pelvic and inguinal regions. However, there have been only two reports of this procedure (this study and [24]).

**Figure 1:**
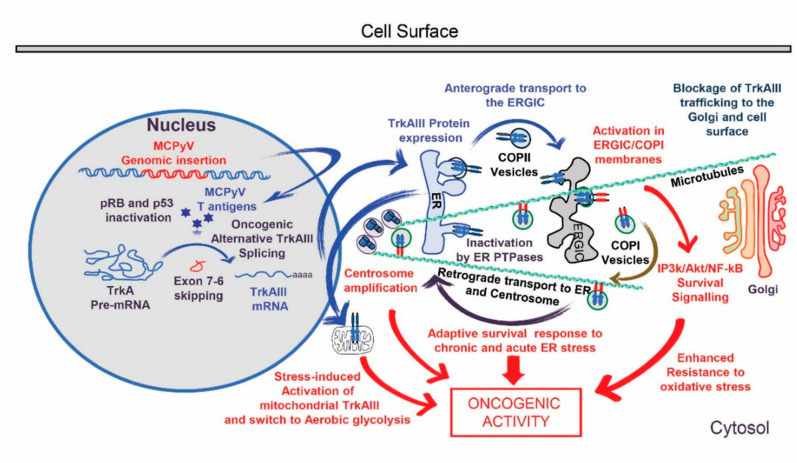
Schematic representation of genomic Merkel cell polyomavirus (MCPyV) insertion, T-antigen expression and induction of oncogenic alternative Δ exon 6–7 TrkAIII splicing in Merkel cell carcinoma (MCC), leading to spontaneous intracellular Δ exon 6–7 TrkAIII activation within endoplasmic reticulum intermediate and COP1 vesicle membrane compartments (ERGIC/COP1), inhibition of anterograde Δ exon 6–7 TrkAIII transport, retrograde-activated Δ exon 6–7 TrkAIII transport back to the endoplasmic reticulum (ER), centrosome and mitochondria, and oncogenic activity characterised by inositol-triphosphate 3-kinase (IP3K)/Akt survival signalling, centrosome amplification, an adaptive survival ER-stress response, enhanced resistance to oxidative stress and stress-induced aerobic glycolysis.

MCC diagnosis, however, continues to carry poor prognosis in spite of these advances, highlighting the need for a further understanding of oncogenic mechanisms involved in this rare tumour type and translation into novel therapeutic strategies. Within this context, in a recent pilot study of MCC FFPE tissues, we reported a novel potential MCPyV oncogenic mechanism, characterised by the promotion of oncogenic alternative Δ exon 6–7 TrkAIII mRNA splicing of the tropomyosin-related tyrosine kinase receptor TrkA [28]. Oncogenic alternative Δ exon 6–7 TrkAIII mRNA splicing was originally identified in advanced-stage primary human neuroblastomas (NBs), results in expression of the Δ exon 6–7 TrkAIII oncoprotein that transforms NIH3T3 mouse embryo fibroblasts and exhibits oncogenic activity in a variety of in-vitro and in-vivo models [29,30]. Furthermore, analysis of potential promoters of alternative Δ exon 6–7 TrkAIII splicing in NB cells identified the polyomavirus simian virus 40 (SV40) large T-antigen [30], raising the possibility that closely related polyomavirus MCPyV T-antigens may also promote alternative Δ exon 6–7 TrkAIII splicing in MCPyV-positive MCCs. This hypothesis was supported in our pilot study [28], which detected a close relationship between MCPyV T-antigen expression and alternative Δ exon 6–7 TrkAIII splicing in FFPE MCC tissues, characterising a new MCC subset and identifying a novel potential MCPyV oncogenic mechanism and therapeutic target (Figure 1). This, however, could not be fully verified due to the poor RNA quality and difficulty in protein extraction from FFPE tissue [28].

In the present study, therefore, we extend our previous report supporting the use of FFPE MCC tissue for MCPyV-positive MCC diagnosis, retrospective study and molecular analysis to confirm the association between MCPyV T-antigen and oncogenic alternative Δ exon 6–7 TrkAIII splicing in fresh nonfixed MCPyV-positive metastatic MCC. We also extend the potential role of this novel oncogenic mechanism from MCC pathogenesis to post-therapeutic relapse and progression and propose that its detection may identify MCC patients who could benefit from novel MCPyV T-antigen and TrkAIII inhibitory therapeutic strategies.

## 2. Results

### 2.1. Clinical Data

**Figure 2:**
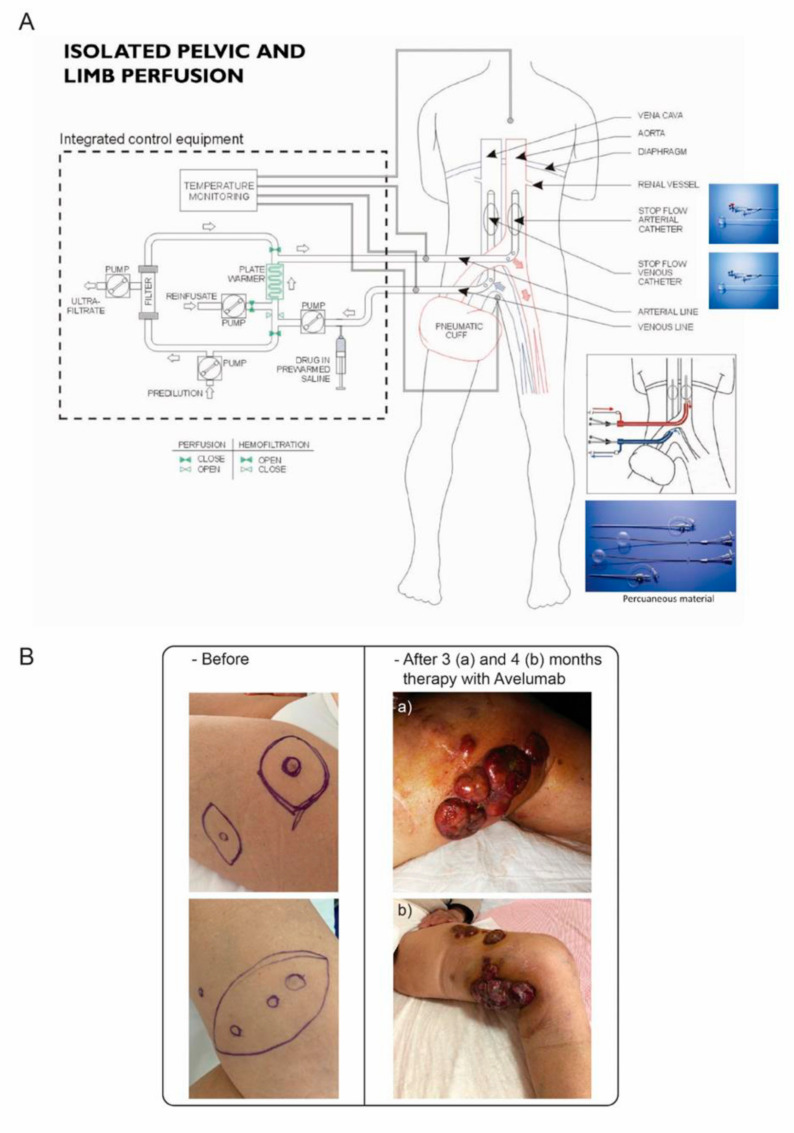
(**A**) Schematic representation of isolated pelvic and limb perfusion with extracorporeal blood circulation controlled by an integrated equipment circuit, complete with chemofiltration unit and apparatus used for open and percutaneous approaches (cartouche). (**B**) Response to avelumab therapy: MCC tumours prior to avelumab therapy (left panels) and following (a) 3 months and (b) 4 months of avelumab therapy (right panels).

From a total of 7175 skin cancers, 12 MCCs (0.17%) spanning the period 2009–2019, histologically diagnosed by immunoreactivity for neural cell adhesion molecule (CD56), cytokeratin 20 and cytokeratin AE1/AE3 were selected from the San Salvatore Hospital Department of Pathology database (L’Aquila, Italy). The median age was 73.5 years (interquartile range (iqr) = 60.5–82.5), and the clinical characteristics of the cohort are reported in Table 1. The median overall survival time following multidisciplinary treatment and from diagnosis was 26 months (iqr = 21–35); 10 patients treated prior to 2019 were dead by February 2020, and 2 patients were still alive at 13 and 5 months from the time of diagnosis, with the disease. All 12 patients underwent surgical excision in multidisciplinary treatment regimes, which included subsequent RT in 2 cases, Melphalan IPLP in 3 cases (Figure 2A), systemic chemotherapy in 2 cases and immune checkpoint inhibitor therapy in 2 cases. One patient, who presented with chronic neutropenia and received concomitant sofosbuvir/daclastavir therapy for HCV infection [24], experienced an unexpected 56-month response to multidisciplinary treatment, consisting of surgical excision followed by melphalan IPLP (Table 2). Approximately one year following the cessation of antiviral therapy, this patient developed thigh recurrences and was treated with avelumab PD-L1 inhibitory monoclonal antibody [14] in accordance with multidisciplinary board suggestions, but failed to respond (Figure 2B).

Two other patients with stage IV distant metastatic disease were subjected to surgical excision combined with IPLP. Both patients experienced brief 3- and 7-months of control of local disease, followed by local progression, and were subsequently treated with systemic chemotherapy but failed to respond (Table 2).

### 2.2. Molecular Data

**Figure 3:**
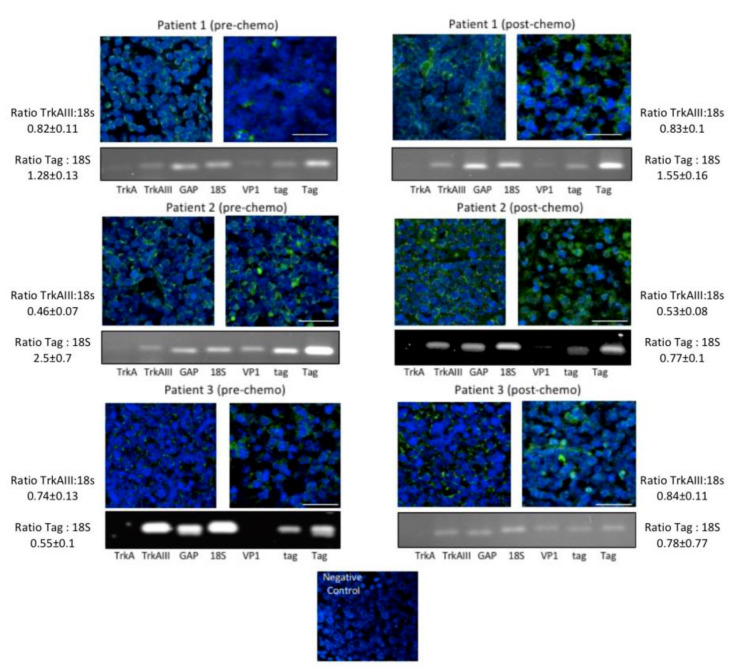
Representative indirect immunofluorescence (IF) micrographs demonstrating immunoreactivity to anti-TrkA and anti-Y490 phosphorylated TrkA antibodies in 5 μm formalin-fixed paraffin-embedded (FFPE) sections of primary MCCs from 3 patients prior to chemotherapy (prechemo) and postchemotherapy (postchemo) recurrent MCCs from the same patients (bar = 50 μm). Negative control represents MCC FFPE tissue from Patient 3 incubated with preimmune rabbit IgG and secondary FITC conjugated anti-rabbit antibody. Under each set is a representative agarose gel demonstrating RT-PCR levels of TrkA, TrkAIII, glyceraldehyde 3-phosphate dehydrogenase (GAP), 18S RNA, MCPyV viral capsid protein (VP1), MCPyV small t-antigen (tag) and MCPyV large t-antigen (Tag) in RNAs purified from 50 μm serial sections of the same FFPE MCC tissues. The densitometric ratios (±SD) of TrkAIII to 18S rRNA (TrkAIII: 18S) and MCPyV Tag to 18S rRNA (Tag: 18S) RT-PCR products are provided for each patient.

RT-PCR detected MCPyV VP-1, small t-antigen and large T-antigen mRNA expression in RNAs from 11 of 12 FFPE MCC tissues, confirming a > 90% MCPyV-positive MCC cohort (Table 2). RNAs exhibiting MCPyV large T-antigen expression also generated sequence-verified RT-PCR products, consistent with alternative Δ exon 6–7 TrkAIII mRNA splicing, that were not detected in the MCPyV T-antigen-negative FFPE MCC (this study and [28]). Indirect IF analysis of MCPyV-positive FFPE MCC tissues also detected IF immunoreactivity for intracellular TrkA protein isoforms and variable immunoreactivity for tyrosine-phosphorylated TrkA isoforms, consistent with potential intracellular Δ exon 6–7 TrkAIII protein expression and activation (Table 3). In an important extension of our previous study, we were fortunate to obtain FFPE MCC tissues from three of the original 12 patients of the cohort, which permitted a comparison of the influence of melphalan IPLP chemotherapy on MCPyV T-antigen and Δ exon 6–7 TrkAIII expression. In this comparison, no significant differences were detected in the mean (± SD) densitometric ratios of MCPyV T-antigen or Δ exon 6–7 TrkAIII to 18S rRNA RT-PCR, employed as a stable internal control for relative RT-PCR analysis (Figure 3), and IF immunoreactivity for phosphorylated TrkA isoform(s) in MCCs that relapsed following melphalan IPLP chemotherapy was, if anything, enhanced rather than reduced (Figure 3), supporting a potential role for this oncogenic mechanism in post-therapeutic disease progression.

**Figure 4:**
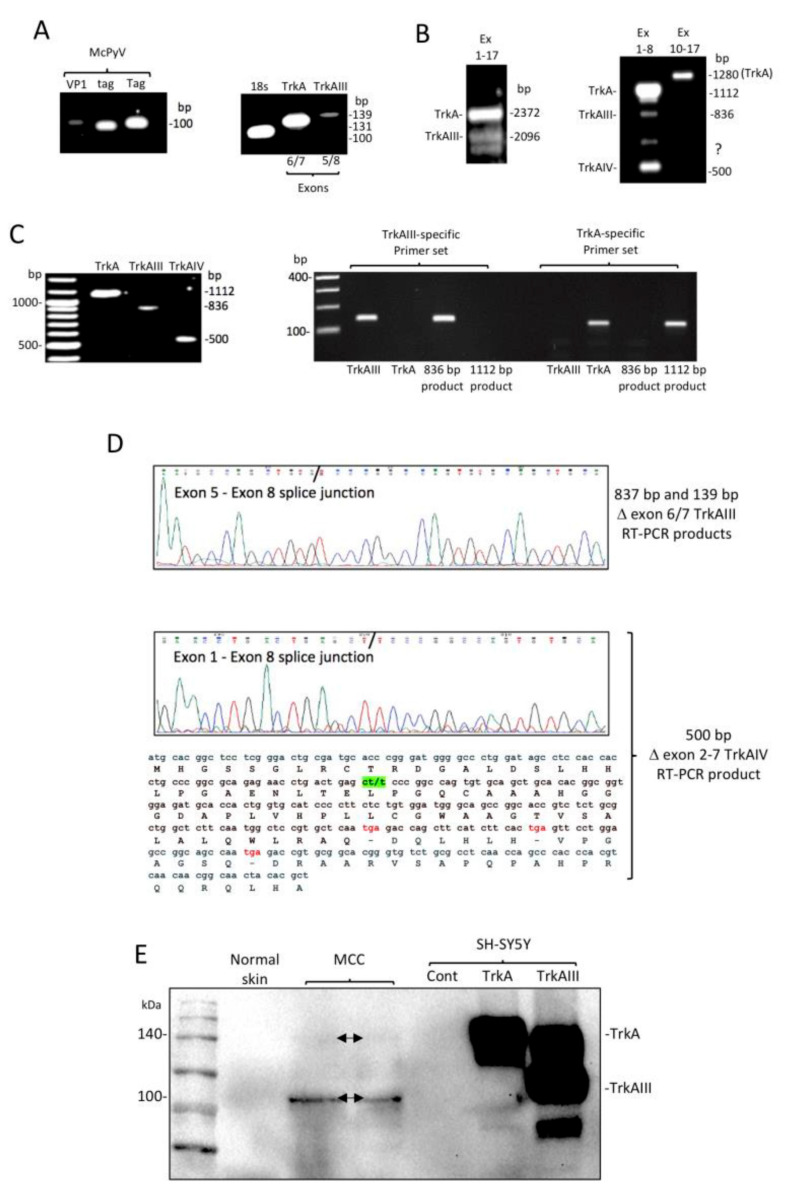
(**A**) RT-PCR demonstrating MCPyV VP1, small t-antigen (tag) and large T-antigen (Tag) (left panel), and 18s rRNA, exon 6 and 7 containing (exons 6/7 TrkA) and noncontaining (exons 5/8, TrkAIII) products generated from metastatic MCPyV-positive MCC RNA. (**B**) RT-PCR demonstrating (left panel) a 2372-bp product expected for full-length fully spliced TrkA and a 2096-bp product expected for Δ exon 6–7 TrkAIII generated from metastatic MCC RNA using primers spanning *TrkA* exons 1 to 17, and (right panel) a 1112-bp product expected for fully spliced TrkA exons 1–8, an 836-bp product expected from Δ exon 6–7 TrkAIII exons 1–8, a yet to be characterised product (?) and the 500-bp product characterised as Δ exon 2-7 TrkAIV, generated from metastatic MCPyV-positive MCC RNA, using primers spanning *TrkA* exons 1 to 8, plus a unique 1280-bp product expected for fully spliced TrkA exons 10–17, generated from metastatic MCPyV-positive MCC RNA using primers spanning *TrkA* exons 10 to 17. (**C**) Gel-purified 1112-bp (fully spliced TrkA), 836-bp (Δ exon 6–7 TrkAIII) and 500-bp (Δ exon 2-7 TrkAIV) *TrkA* exon 1 to 8 RT-PCR products (left panel), and (right panel) RT-PCR confirmation of Δ exon 6–7 TrkAIII and TrkA identity of TrkA and D exon 6–7 TrkAIII control cDNAs, gel-purified 1112-bp TrkA exon 1–8 and 836-bp Δ exon 6–7 TrkAIII RT-PCR products, using TrkA and Δ exon 6–7 TrkAIII-specific primers. (**D**) Sequence confirmation of the novel Δ exon 6–7 TrkAIII exon 5/8 splice junction in the gel-purified 836-bp Δ exon 6–7 TrkAIII exon 1–8 RT-PCR product (3rd panel), and confirmation of the novel exon 1/8 splice junction (green) in the 500-bp Δ exon 2–7 TrkAIV exon 1–8 RT-PCR product (4th panel), plus a section of nucleotide and potential amino acid sequence for Δ exon 2–7 TrkAIV demonstrating frame-shift induced tga stop codons (red). (**E**) Western blot demonstrating 140 kDa and 100 kDa (arrows) TrkA immunoreactive species in protein extracts from the MCPyV-positive metastatic MCC (MCC) but not in a normal skin extract (100 μg loads), compared to 140 kDa fully spliced TrkA and 100 kDa Δ exon 6–7 TrkAIII in stable TrkA and Δ exon 6–7 TrkAIII transfected SH-SY5Y cell extracts but not in stable empty pcDNA vector-transfected SH-SY5Y cell extract negative control (Cont; 20 μg loads).

In our previous study [28], the relationship between MCPyV and oncogenic alternative Δ exon 6–7 TrkAIII splicing, detected in FFPE MCC tissues, could not be fully verified due to poor RNA quality and difficulty in protein extraction [31]. Here, we present evidence that confirms this relationship in tissue from fresh nonfixed MCPyV-positive MCC metastasis that subsequently became available from one patient in this cohort. RT-PCR of undegraded RNAs from this metastatic MCC detected: (i) MCPyV VP1, small t-antigen and large T-antigen mRNA expression confirming an MCPyV-positive diagnosis (Figure 4A); (ii) a 2372-bp (base pair) product expected for full length fully spliced TrkA and a 2096-bp product expected for full length alternatively spliced Δ exon 6–7 TrkAIII, using primers spanning *TrkA* exons 1 to 17; (iii) a 1112-bp product expected for fully spliced exon 1-8 TrkA and an 836-bp product expected for Δ exon 6–7 TrkAIII, using primers spanning *TrkA* exons 1 to 8; (iv) a 139-bp product expected for Δ exon 6–7 TrkAIII using the TrkAIII-specific primer set; (v) a single 1280-bp product expected for fully spliced exons 10 to17 TrkA, using primers spanning *TrkA* exons 10–17 (Figure 4B). The 1112-bp and 836-bp exon 1–8 RT-PCR products were gel-purified and further characterised as representing fully spliced TrkA and alternatively spliced Δ exon 6–7 TrkAIII, respectively, by RT-PCR using TrkA and Δ exon 6–7 TrkAIII-specific primers (Figure 4C) and by detection of exons 6, 7 and 8 sequences in the 1112-bp fully spliced TrkA product (not shown) and the novel exon 5–8 splice junction in the 836-bp Δ exon 6–7 TrkAIII product (Figure 4D), identical to the original TrkAIII sequence (29) deduced from TrkAI splice variant reference sequence NM_001012331.2. Additional RT-PCR products generated using primers spanning TrkA exons 1 to 8 included a major 500-bp product (Figure 4B), which was also gel-purified (Figure 4C) and sequence-characterised as a novel alternative Δ exon 2-7 TrkAIV splice variant exhibiting cassette exons 2 to 7 skipping, coding for 3 frame-shift-induced stop codons initiating at 40 codons downstream of the novel exon 1–8 splice junction (Figure 4C,D).

In addition to RT-PCR, Western blots with an antibody that recognises both fully-spliced TrkA and Δ exon 6–7 TrkAIII [28,29] detected a 100-kDa immunoreactive species in protein extracts (100 μg) from the fresh metastatic MCC, not detected in an extract of normal skin (100 μg), of identical size to 100-kDa Δ exon 6–7 TrkAIII detected in stable Δ exon 6–7 TrkAIII transfected SH-SY5Y cell extracts but not in either stable TrkA-transfected SH-SY5Y cell extracts, which contained 140-kDa fully spliced TrkA or in stable empty pcDNA vector-transfected SH-SY5Y cell extracts used as a negative control (20 μg) [29] (Figure 4E), consistent with Δ exon 6–7 TrkAIII protein expression in the fresh MCPyV-positive metastatic MCC.

## 3. Discussion

Rare, highly aggressive neuroendocrine MCC skin cancers invariably carry a poor prognosis, which combined with a general lack of specific multidisciplinary therapeutic consensus for the treatment of advanced locoregional and stage IV disease, highlights the great need for improved understanding of the oncogenic mechanisms involved in MCC pathogenesis, post-therapeutic relapse and progression, and translation into novel therapeutic strategies.

Here, we present an extension of a previous pilot study [28] that confirms the importance of FFPE MCC tissues as a source of rare tumour material for MCPyV-positive MCC diagnosis, retrospective study and molecular investigation of novel potential oncogenic mechanisms. We also confirm the association between MCPyV gene expression and oncogenic alternative Δ exon 6–7 TrkAIII splicing in a fresh nonfixed metastatic MCC and extend the potential roles of MCPyV-driven oncogenic alternative Δ exon 6–7 TrkAIII splicing in MCC from pathogenesis to postchemotherapeutic relapse and progression. Whilst this genetic association cannot currently be considered causative, our observations focus attention on the therapeutic possibility of targeting this novel potential oncogenic mechanism in MCC.

The importance of FFPE tissues as a source of rare tumour material for retrospective study and molecular analysis is contrasted by the poor quality of RNA and difficulty in extracting proteins from FFPE tissues, making analysis and interpretation of gene expression difficult [31]. Previously, we reported RT-PCR that MCPyV T-antigen mRNA expression was frequently associated with oncogenic alternative Δ exon 6–7 TrkAIII splicing in FFPE MCC tissues, characterising a new MCPyV-positive MCC subgroup and identifying a novel potential MCPyV oncogenic mechanism [28]. This was based upon detecting small (<200 bp) sequence-characterised Δ exon 6–7 TrkAIII RT-PCR products and by indirect IF evidence supporting potential intracellular Δ exon 6–7 TrkAIII TrkA protein expression and activation. However, this could not be fully verified by additional RT-PCR or Western blotting due to problems with FFPE tissues [28]. Here, we present evidence that confirms the association between *MCPyV* gene expression and oncogenic alternative Δ exon 6–7 TrkAIII splicing in a fresh nonfixed MCPyV-positive MCC metastasis that became available from one patient in the cohort, extending the possible roles of this potential oncogenic mechanism to metastatic disease. RT-PCR of this metastatic MCPyV-positive MCC detected expected products for both full-length fully spliced TrkA and full-length alternatively spliced Δ exon 6–7 TrkAIII, with the 1112-bp exon 1–8 fully spliced TrkA and 836-bp exon 1–8 and 139-bp Δ exon 6–7 TrkAIII RT-PCR products confirmed by DNA sequence comparison to the TrkAI splice variant reference sequence NM_001012331.2, confirming alternative Δ exon 6–7 TrkAIII mRNA splicing. Furthermore, the unique 1280-bp *TrkA* exons 10 to 17 RT-PCR product, expected for fully spliced TrkA, confirms that alternative TrkA splicing in this metastatic MCPyV-positive MCC involved TrkA exons 1–8 but not exons 10–17. Furthermore, Western blots also detected a 100-kDa TrkA isoform, consistent with Δ exon 6–7 TrkAIII protein, in protein extracts from the metastatic MCC, which was not detected in a normal skin extract and was of an identical molecular size to the 100-kDa Δ exon 6–7 TrkAIII expressed by stable Δ exon 6–7 TrkAIII-transfected SH-SY5Y cells compared to 140-kDa fully spliced TrkA expressed by stable TrkA-transfected SH-SY5Y cells pcDNA-transfected SH-SY5Y cells (negative control) [29]. An additional and prominent RT-PCR product was also detected in metastatic MCC RNA and was characterised as a novel Δ exon 2–7 TrkAIV splice exhibiting cassette exon 2–7 skipping, resulting in the introduction of 3 frame shift-induced stop codons downstream of the novel exon 1–8 splice junction. We are currently investigating whether this is expressed as a truncated protein and/or exhibits long noncoding RNA functions.

In addition, we also report comparative molecular and indirect IF analysis of FFPE MCC tissues from three patients, obtained prior to and following melphalan- or platinum-based chemotherapy. This comparison did not detect any reductions in the constitutive pretreatment levels of either MCPyV large T-antigen mRNA expression, Δ exon 6–7 TrkAIII mRNA expression or TrkA and phosphorylated TrkA isoform(s) IF immunoreactivity in tumours that relapsed following treatment. Although this does not prove a causative role for Δ exon 6–7 TrkAIII splicing in treatment resistance, we have previously shown that Δ exon 6–7 TrkAIII enhances NB cell resistance to a variety of agents, including cisplatin [30], suggesting the possibility that, in addition to playing potential oncogenic roles in MCC pathogenesis and metastatic progression, alternative Δ exon 6–7 TrkAIII splicing may also promote therapeutic resistance and postchemotherapeutic relapse in this tumour type, further focusing attention on the therapeutic possibility of targeting this potential mechanism in MCPyV-positive MCC.

Of additional interest, one patient with stage IIIB MCPyV-positive MCC experienced an unexpected 56-month response duration following surgical tumour excision and IPLP locoregional therapy [24]. This patient presented with chronic neutropenia and received concomitant sofosbuvir/daclastavir therapy for Hepatitis C infection, suggesting that sofosbuvir/daclastavir therapy may have inadvertently and indirectly inhibited the oncogenic activity MCPyV, rendering disseminated MCC cells that survived locoregional IPLP dormant for the duration of antiviral therapy. This patient presented with relapsed disease approximately 1 year after the suspension of antiviral therapy and was treated with avelumab PD-L1 checkpoint inhibitor but failed to respond (Figure 2B) and subsequently died, suggesting that immune checkpoint inhibitor efficacy should also be re-evaluated in patients presenting with altered immune/inflammatory responses [32] and in patients with stage IIIB, in addition to stage IV, disease.

Limitations of this study include the small retrograde sample size explained by the relative rarity of MCC, the poor quality of RNAs and the impracticability of protein extraction from FFPE tissues, limiting accurate gene expression evaluation, addressed in this study using a fresh nonfixed MCPyV-positive MCC metastasis, and the fact that this is a single-centre study due to the lack of specialised centres that perform surgery with IPLP and provide tissues for molecular analyses.

## 4. Patients and Methods

### 4.1. Patients

This STROCSS-criteria-compliant [33] retrospective cohort study was approved by the Ethics committee of ASL n.1, Abruzzo, Italy (Chairperson: G. Piccioli; protocol number10/CE/2018 n.1419). All patients provided fully informed written consent, including the publication of scientific images. The 12 MCC skin cancers in this study were histologically diagnosed from 2009 to 2019, selected from the database of the Department of Pathology San Salvatore Hospital, L’Aquila, and the clinical characteristics are reported in Table 1 and represent the same patient cohort reported previously in our pilot study [28].

### 4.2. Clinical Methods

Multidisciplinary treatments included surgical oncological excision in all 12 cases, followed by RT in 2 patients, locoregional IPLP chemotherapy in 3 patients, systemic chemotherapy in 2 patients, and immune checkpoint inhibitor therapy in 2 patients. Surgical and percutaneous IPLP procedures (Figure 2A) were performed under general anaesthesia and are detailed elsewhere [24,26,27]. Melphalan was used at a dose of 30 mg/m^2^.

### 4.3. Molecular Methods

MCPyV VP-1, large T-antigen and small t-antigen mRNA expression, as an index of MCPyV infection and genomic integration, and TrkA isoform mRNA expression were assessed by RT-PCR, using gene-specific primers to generate RT-PCR products of <200-bp for MCPyV VP1, small t-antigen, large T-antigen, exon 6 and 7 containing TrkA mRNAs, and exon 6 and 7 skipped Δ exon 6–7 TrkAIII mRNA and 18S rRNA, as described previously [28]. Primer selection was dictated by the reported poor quality of degraded RNAs purified from FFPE tissues [31]. For semiquantitative densitometric analysis, triplicated RT-PCR products for TrkAIII and 18S rRNA were amplified from individual tissue samples, compared within the same 1% agarose gel, digitally photographed and analysed using Image J software (ImageJ bundled with Java 1.8.0_172) [34]. Intergel comparisons were made using common 18S rRNA RT-PCR product and DNA ladder standards, as previously described [28].

Furthermore, due to limited quantity and difficulty in extracting proteins from FFPE MCC tissues, evidence for TrkA protein isoform(s) expression and activation (TrkA Y490 tyrosine phosphorylation) was approximated in serial FFPE MCC tissue by indirect IF using TrkA carboxyl terminal-domain (C14, Santa Cruz Inc, Santa Cruz, CA, USA) and Y490 tyrosine-phosphorylated TrkA (pY490 TrkA, Cell Signaling, CA, USA) antibodies, as previously described [28,29].

The effect of chemotherapy on MCPyV, TrkA and Δ exon 6–7 TrkAIII mRNA expression and on TrkA- and Y490-phosphorylated TrkA isoform IF immunoreactivity was assessed by comparing FFPE tissues from the MCCs obtained prechemotherapy and in postchemotherapeutic recurrent MCCs from the same individual.

A fresh metastatic MCPyV-positive MCC tissue sample, surgically removed from one patient in the cohort, was used to extract good quality RNA for RT-PCR reactions, as previously described [29], in order to provide evidence of MCPyV gene VP-1, small t-antigen and large T-antigen mRNA and 18S rRNA expression, using previously described primer sets [28], and for fully spliced TrkA, Δ exon 6–7 TrkAIII and additional TrkA isoforms, using primer sets spanning TrkA exons 1 to 17 (forward primer 5′-ATGCTGCGAGGCGGACGGCGC-3′ and reverse primer 5′-CTAGCCCAGG ACATCCAGGTA-3′), TrkA exons 1 to 8 (forward primer 5′-ATGCTGCGAGGCGGACGGCGC-3′ and reverse primer 5′-GGAGGCCTGGCCGAAGGGGTT-3′), TrkA exons 10–17 (forward primer 5′-AACCCCTTCGGCCAGGCCTCC-3′ and reverse primer 5′-CTAGCCCAGGACATCC AGGTA-3′), TrkA exon 6–7 (forward primer 5′- AGCCACGGTGATGAAATCTGGGGGTC T-3′ and reverse primer 5′-TTGACCTGAACAGAGACCTCTGC-3′) and the Δ exon 6–7 TrkAIII-specific primer set (forward primer 5′-AATGCCAGCTGTGTCCCG-3′ and reverse primer 5′-TGGTC TCATTGAGCACGGAG-3′) [35]. Gel extraction of ethidium bromide-stained RT-PCR products was performed using a QIAquick gel extraction kit, as described by the manufacturer (Qiagen, Milan, IT). Protein extracts from the fresh nonfixed MCC were subjected to Western blotting using an anti-TrkA antibody (C14, Santa Cruz Inc., Santa Cruz, CA, USA) that recognises both fully spliced TrkA and alternatively spliced Δ exon 6–7 TrkAIII [29] and were compared to fully spliced TrkA and Δ exon 6–7 TrkAIII proteins (5 μg), extracted from stable-transfected TrkA and Δ exon 6–7 TrkAIII SH-SY5Y cells [29].

### 4.4. Statistical Analysis

Statistical analyses were performed using STATA software (version 14, Stata Corp, College Station, TX, USA). For clinical data, continuous variables are expressed as median with interquartile range (iqr). For molecular data, continuous variables are displayed as the mean ± standard deviation (SD). For both categories, qualitative data are expressed as frequency or percentage.

## 5. Conclusions

In conclusion and in pursuit of future therapeutic improvements for the treatment of MCC, we (i) stress the importance of multidisciplinary treatment, including surgical excisions and repetitive locoregional chemotherapy, not only for optimising local disease control [36] and enhancing overall survival [17,18,19,20,21,22,23,24,25] but also for palliative care, considering the psychological importance of surgical tumour removal; (ii) suggest that sofosbuvir/daclastavir therapy should be investigated as a potential indirect inhibitor of MCPyV oncogenic activity; (iii) confirm that oncogenic alternative Δ exon 6–7 TrkAIII splicing not only occurs in MCPyV-positive MCC but also in metastatic disease, focusing attention on this novel potential MCPyV oncogenic mechanism as a possible therapeutic target. We propose that detection alternative Δ exon 6–7 TrkAIII identifies an MCC subgroup that may benefit from novel inhibitors of MCPyV T-antigen and Δ exon 6–7 TrkAIII expression or clinically approved Trk kinase inhibitors such as larotrectinib and entrectinib, known to inhibit mutation and deletion-activated TrkA oncogenes and to elicit durable responses in a wide range of advanced-stage TrkA-fusion oncogene-driven cancers [37,38,39], supporting the call for a large multicentre study.

## Figures and Tables

**Table 1:** Clinical characteristics of Merkel cell carcinoma (MCC) patients.

Clinical Characteristics	N (%)
**Gender**	
Female	7 (58.3)
Male	5 (41.7)
**Age (Years)**	
≤65	4 (33.3)
>65	8 (66.7)
**Primary Location**	
Head and/or Neck	2 (16.7)
Trunk	4 (33.3)
Extremities	6 (50.0)
**Stage (AJCC 2019) at Molecular Analysis**	
I	2 (16.7)
II A	4 (33.3)
II B	1 (8.3)
III B	3 (25.0)
IV	2 (16.7)
**Multidisciplinary Treatment**	
Surgical excision	12 (100.0)
Radiation therapy	2 (16.7)
Melphalan IPLP	3 (25.0)
Systemic chemotherapy	2 (16.7)
Immune checkpoint inhibitors	2 (16.7)
**Status**	
Dead	10 (83.3)
Alive	2 (16.7)

**Table 2:** Clinical characteristics of three advanced-stage MCC patients submitted for locoregional chemotherapy during multidisciplinary treatment.

(A) Patient Id (B) Primary Site (C) Recurrence Location/Age (Years)/Stage	(A) Previous Therapy (B) Concomitant Disease (Therapy)	1st Treatment Distant Disease Status	2nd Treatment Distant Disease Status	3rd Treatment Distant Disease Status	(A) Concomitant Therapy Before Progression (B) PFS from 1st Locoregional Treatment (C) Progression Site (D) Therapy at Progression	Censor (February 2020) OS from 1st Locoregional Treatment
**(A)** 1 **(B)** Calcaneal region **(C)** Groin and limb /73/IIIB (pathological)	**(A)** WSE; SNB; CLND Chronic Neutropenia **(B)** Hepatitis C (Sofosbuvir/ Daclastavir)	WSE (10 nodules >1 cm diameter) IPLP	WSE (3 nodules <1 cm diameter) IPLP	WSE (1 nodule <1 cm diameter) IPLP	**(A)** Sofosbuvir/ Daclastavir (24 months) **(B)** 56 months **(C)** Locoregional **(D)** Avelumab	Dead 59 months
**(A)** 2 **(B)** Anterior abdomen **(C)** Pelvis and limb plus distant/58/IV (clinical)	**(A)** WSE and RT	WSE (7 nodules >2 cm diameter) IPLP SDi in bone metastases	WSE (5 nodules >2 cm diameter) IPLP SDi in bone metastases	WSE (4 nodules >2 cm diameter) IPLP SDi in bone metastases	**(B)** 7 months **(C)** Locoregional and distant **(D)** Platinum-based systemic chemotherapy until dead	Dead 12 months
**(A)** 3 **(B)** Gluteal region **(C)** Pelvis and limb plus distant/75/IV (clinical)	**(A)** WSE **(B)** Heart arrhythmia (Coumadin); Glaucoma (Propanolol)	WGSE/CLND (5 metastatic lymph nodes) IPLP SDi in lung, liver and brain metastases	IPLP alone SDi in lung, liver and brain metastases		**(A)** Coumadin/ Propanolol **(B)** 3 months **(C)** Locoregional and distant **(D)** Platinum-based systemic chemotherapy for 23 months	Dead 30 months

PFS, progression free survival; OS, overall survival; IPLP, isolated pelvic limb perfusion; WSE, wide surgical excisions; WGSE, wide gluteal surgical excision; SNB, sentinel lymph node biopsy; CLND, complete lymph node dissection; SDi, stable disease; RT, radiotherapy.

**Table 3:** Molecular characteristics of MCC patients.

MCC Molecular Characteristics.	N (%)
***MCPyV* Gene Expression**	
Yes	11 (91.7)
No	1 (8.3)
**MCPyV Large T-Antigen**	
Present	11 (91.7)
High	10 (83.4)
Moderate	1 (8.3)
Negative	1 (8.3)
**TrkA Expression**	
High	1 (8.3)
Moderate	4 (33.3)
Low	7 (58.4)
**TrkAIII Expression**	
High	8 (66.7)
Moderate	3 (25.0)
Low	1 (8.3)
**Y490 Phosphorylated TrkA/TrkAIII IF**	
High.	6 (50.0)
Moderate	2 (16.7)
Low	1 (8.3)
Negative	3 (25.0)

## Abbreviations

bp	base pair
CLND	complete lymph node dissection
CD56	neural cell adhesion molecule
CT	computerised tomography
EpCAM	epithelial cell adhesion molecule
FDA	food and drug administration
FFPE	formalin-fixed paraffin-embedded
IF	indirect immunofluorescence
IgG1	immunoglobulin G1
INFγ	interferon gamma
ILI	isolated limb infusion
ILP	isolated limb perfusion
IPLP	isolated pelvic and limb perfusion
MCCs	Merkel cell carcinomas
MCPyV	Merkel cell polyomavirus
NB	neuroblastoma
NCCN	National Comprehensive Cancer Network
NIH3T3	mouse embryonic fibroblast cells
OS	overall survival
PD-1	programmed cell death protein 1
PD-L1	programmed death-ligand 1
RNAs	ribonucleic acids
RT	radiotherapy
RT-PCR	real-time polymerase chain reaction
SNB	sentinel lymph node biopsy
SD	standard deviation
SV40	simian vacuolating 40 polyomavirus
TNF	tumour necrosis factor
TrkA	tropomyosin-related kinase A
USA	United States of America
UV	ultraviolet
VEGFR	vascular endothelial growth factor receptor
WSE	wide surgical excisions
WGSE	wide gluteal surgical excision
SDi	stable disease
PFS	Progression-free survival
